# The Value of Convolutional Neural Network-Based Magnetic Resonance Imaging Image Segmentation Algorithm to Guide Targeted Controlled Release of Doxorubicin Nanopreparation

**DOI:** 10.1155/2021/9032017

**Published:** 2021-07-26

**Authors:** Hujun Liu, Hui Gao, Fei Jia

**Affiliations:** Department of Pharmaceuticals, Affiliated Hospital of Yan'an University, Yan'an 716000, Shaanxi, China

## Abstract

There was an investigation of the auxiliary role of convolutional neural network- (CNN-) based magnetic resonance imaging (MRI) image segmentation algorithm in MRI image-guided targeted drug therapy of doxorubicin nanomaterials so that the value of drug-controlled release in liver cancer patients was evaluated. In this study, 80 patients with liver cancer were selected as the research objects. It was hoped that the CNN-based MRI image segmentation algorithm could be applied to the guided analysis of MRI images of the targeted controlled release of doxorubicin nanopreparation to analyze the imaging analysis effect of this algorithm on the targeted treatment of liver cancer with doxorubicin nanopreparation. The results of this study showed that the upgraded three-dimensional (3D) CNN-based MRI image segmentation had a better effect compared with the traditional CNN-based MRI image segmentation, with significant improvement in indicators such as accuracy, precision, sensitivity, and specificity, and the differences were all statistically marked (*p* < 0.05). In the monitoring of the targeted drug therapy of doxorubicin nanopreparation for liver cancer patients, it was found that the MRI images of liver cancer patients processed by 3D CNN-based MRI image segmentation neural algorithm could be observed more intuitively and guided to accurately reach the target of liver cancer. The accuracy of targeted release determination of nanopreparation reached 80 ± 6.25%, which was higher markedly than that of the control group (66.6 ± 5.32%) (*p* < 0.05). In a word, the MRI image segmentation algorithm based on CNN had good application potential in guiding patients with liver cancer for targeted therapy with doxorubicin nanopreparation, which was worth promoting in the adjuvant treatment of targeted drugs for cancer.

## 1. Introduction

Liver cancer refers to cancer that occurs in the liver and is one of the most common tumors in China [1, 2]. According to the data released by the National Cancer Center, it was estimated that there would be 466,100 new cases and 422,100 deaths in 2018, with a very high proportion of deaths after onset. Moreover, the incidence of this disease has a trend of gradual increase with age. As for the disease distribution data, the incidence of liver cancer in rural areas is higher than that in urban areas, and the incidence of liver cancer in males is also higher than that in females [3–5]. Patients with early liver cancer usually do not have any symptoms. As the disease progresses, they may experience loss of appetite, abdominal pain and bloating, unexplained weight loss, and yellowish skin and sclera [6]. Clinically, diagnostic methods for liver cancer include imaging examinations (dynamic contrast-enhanced magnetic resonance imaging (MRI), dynamic contrast-enhanced computed tomography (CT), and selective hepatic arteriography), the alpha-fetoprotein (AFP) indicators in blood-drawn results, and liver lesion tissue biopsy. Among them, the advantage of MRI imaging in the diagnosis of liver cancer lies in its high sensitivity, which can display the tissue structure of liver cancer more clearly than CT examination. Besides, MRI examination adds coronal and sagittal examination based on the cross section, which can reduce the interference of artifacts to a certain extent [7–9]. There are differences in treatment methods for different stages and different types of liver cancer. The currently available treatment methods include local treatment (surgery, ablation, embolization, and radiotherapy) and system therapy (targeted therapy, immunotherapy, and chemotherapy) [10, 11].

In recent years, the algorithm research of convolutional neural network (CNN) in the field of MRI image segmentation has achieved remarkable results. It has been verified by experiments in the clinical diagnosis of nasopharyngeal cancer, breast cancer, spinal metastatic tumor, brain tumor, and other diseases, showing good segmentation effect on MRI images and greatly improving the diagnostic conformance rate of related diseases [12, 13]. Targeted therapy has become a hot topic in the field of cancer therapy because of its advantages such as less trauma, low toxicity, and good selectivity. Clinically, MRI images are mainly applied to observe and guide the release of targeted drugs at specific targets in vivo. Among them, MRI is currently a very valuable method for guiding the release of targeted drugs in the process of targeted therapy for cancer patients. Unfortunately, the observation effect of routine MRI imaging on the targeted controlled release of nanoprepared drugs is not ideal, so it needs to be further analyzed by other methods, such as image segmentation algorithm based on CNN [14]. Based on this, it was hoped that the MRI image segmentation algorithm based on CNN could be applied to the guidance and monitoring of drug release in targeted therapy for liver cancer patients in this study. As an antitumor antibiotic, doxorubicin can inhibit the synthesis of ribonucleic acid (RNA) and deoxyribonucleic acid (DNA) and has the strongest inhibitory effect on RNA. It has a broad antitumor spectrum and has an effect on a variety of tumors. It is a cycle nonspecific drug, which has a killing effect on tumor cells of various growth cycles [15], and its nanopreparations are commonly used drugs for targeted therapy of liver cancer [16]. Therefore, doxorubicin nanopreparation would be selected as the target drug release research object in this study.

To sum up, the use of CNN technology to optimize MRI images to assist physicians in observing and controlling the release of targeted drugs has become a hot topic for scholars. Based on this, an end-to-end neural network architecture was designed in this study based on the fully CNN, which was applied to MRI image analysis of 40 liver cancer patients. The receiver operating characteristic (ROC) curve was employed to comprehensively evaluate the application value of MRI image analysis based on CNN in guiding the targeted controlled release of doxorubicin nanopreparation.

## 2. Materials and Methods

### 2.1. Research Objects

In this study, 60 liver cancer patients admitted to the hospital were selected as the research objects, including 41 males and 39 females. All patients were 39–73 years old, with an average age of 57.5 ± 4.6 years. What is more, the male patients were 54.3 ± 5.2 years old on average and the average age of the female patients was 59.4 ± 4.1 years. Among them, 38 had hepatocellular carcinoma, 13 had intrahepatic cholangiocarcinoma, 4 had intrahepatic angiosarcoma, and 5 had hepatoblastoma. In this study, all patients were rolled randomly into two groups and were examined with MRI scans. The experimental group received a CNN-based MRI image segmentation algorithm for MRI image processing and analysis, while the routine artificial MRI image analysis method was applied to the control group. This study was approved by the Ethics Committee of the hospital, and the patients and their family members included in the study were all informed and signed informed consent forms.

The criteria for inclusion were defined to include patients who did not receive surgery, radiotherapy, and chemotherapy before the experiment, had complete basic clinical data, and had clear MRI image data before surgery. The criteria for exclusion were defined to include patients who were combined with distant metastasis, had an intolerance to doxorubicin preparations, were accompanied with other malignant tumors, and had many MRI image artifacts in their images with poor quality.

### 2.2. Preparation of Doxorubicin Nanopreparation and Targeted Controlled Release Process

The doxorubicin nanopreparation used in this study was composed of nonionic surfactant vesicle (niosomes, N) encapsulated doxorubicin (content: 99.5%, Wuhan Dongkangyuan Technology Co., Ltd.). Besides, the used reagents included triethylamine, span, cholesterol, chloroform, and phosphate buffer saline (PBS).

The preparation process of doxorubicin nanopreparation was as follows:

- Hydrochloric acid (HCl) doxorubicin was dissolved in double-distilled water, which was added with a quantitative amount of triethylamine in a ratio of 1 : 2 for neutralization, and then, there was the freeze-drying treatment.
- Preparation of nonionic surfactant vesicles: first, span, cholesterol, and freeze-dried doxorubicin were dissolved in chloroform, and the mixture was placed on a rotary evaporator to evaporate the organic solvents. Then, it was for vacuum overnight. Next, 10 mL of PBS buffer with a pH of 7.2 was shaken for 15 minutes at 60°C in a water bath to remove the membrane to form a nonionic surfactant vesicle suspension. Finally, the suspension was treated by 50 times continuous and intermittent ultrasound (working time: 2 seconds, interval time: 3 seconds, and power: 200 W) and then separated by a triple filter membrane (0.8 *μ*m, 0.4 *μ*m, and 0.2 *μ*m).
- A laser diffraction particle size analyzer was adopted to determine the particle size of the doxorubicin nanopreparation, and the external morphological characteristics of the doxorubicin nanopreparation were observed under a projection electron microscope.

### 2.3. Establishment of MRI Image Segmentation Algorithm Based on Fully CNN

**Figure 1:**
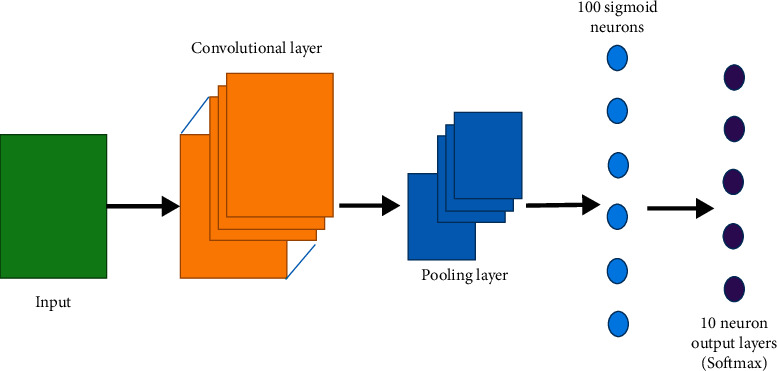
Diagram of the traditional CNN model.

The deep learning technology was employed to automatically segment MRI images, which had great application value in clinical tumor diagnosis and could greatly reduce the degree of dependence on the subjective judgment of physicians. In the traditional CNN structure, a convolutional layer, a pooling layer, and a full-connected layer were mainly included. The neural unit layers of various functions were stacked on each other to form deep CNN, as shown in Figure 1.

The calculation equation of any one of the convolutional layer feature maps can be expressed as follows:

(1) $$\begin{array}{c} H_{x}=g\left(M_{x}*y\right), \end{array}$$

where *x* stands for the *x*-th neuron of a certain convolutional layer, *H*_*x*_ represents the *x*-th feature map calculated by convolution, and *y* means the input image. The weight of the *x*-th neuron can be expressed as *M*_*x*_, *∗* is a 2D convolution operator, which could be employed to calculate the inner product of pixels and weights in the filter window, and *g*() represents a nonlinear activation function.

The maximum pooling equation can be expressed as the following:

(2) $$\begin{array}{c} H_{xab}=\underset{\left(m,n\right)\in D_{ab}}{\max}y_{xmn}. \end{array}$$

where *H*_*xab*_ represents the pooling operation related to the feature map and *y*_*xmn*_ indicates the element at the pooling area *D*_*ab*_. The last layer of the alternating connection between the convolution layer and the pooling layer was connected to the full-connected layer by establishing the connection between features and labels. The activation function of the full-connected layer was commonly expressed by Softmax function, and its definition is as follows:

(3) $$\begin{array}{c} Q\left(s_{z}\right)=\frac{\exp\left(s_{z}^{R}\right)}{\sum_{b=1}^{Z}\exp\left(s_{b}^{R}\right)}. \end{array}$$

In equation (3), *z* represents the target classification number, *S*_*z*_^*R*^ means the predicted value of the *z*-th category, and *s*_*z*_ stands for the *z*-th category. The Softmax function output the probability that the sample belonged to each category, and the sum of the probabilities was 1.

To observe and investigate the targeted controlled release process of nanopreparations, 3D CNN was introduced in this study to make up for the defect that 2D CNN could not obtain timing information in video images. The mathematical equation for 3D convolution is shown in the following equation:

(4) $$\begin{array}{c} u_{ab}^{ij}=g\left(f_{ab}+\sum_{t}\sum_{m=0}^{E_{a}-1}\sum_{n=0}^{F_{a}-1}v_{abt}^{mn}u_{\left(a-1\right)t}^{\left(i+m\right)\left(j+n\right)}\right), \end{array}$$

where *u*_*ab*_^*ij*^ stands for the eigenvalue of the position (*i*, *j*) on the *b*-th feature map in the *a*-th layer neural network, *g*() represents the activation function, and *f*_*ab*_ indicates the bias term. What is more, *t* expresses the feature map index of the layer connected to the current feature map, *v*_*abt*_^*mn*^ represents the weight value, and *E*_*a*_, *F*_*a*_ means the height and width of the convolution kernel in turn, respectively. Bias and weights needed to be determined through training, and other values could be preset in advance.

The maximum pooling operation of 3D convolution can be expressed as follows:

(5) $$\begin{array}{c} P=\left[s_{1}^{a},s_{2}^{a},s_{3}^{a},\ldots ,s_{x}^{a}\right]\in L^{Q\times W\times E\times R}, \end{array}$$

where *R* represents the number of feature spaces, (*Q*, *W*, *E*) stands for the size of the feature map, and *s*_*x*_^*a*^ means the *x*-th feature output map of the *a*-th convolutional layer. The purpose of the 3D convolution maximum pooling operation was to calculate the maximum value in the feature cube.

To reduce the overfitting degree of models and improve their generalization ability in the calculation process of CNN, regularization mode was introduced to optimize the training model. The regularization mode Dropout was adopted in this study. Besides, Dropout was to randomly discard the output values of a layer of neurons. The whole Dropout process was equivalent to taking the average of many different neural networks, while different networks produced different overfitting. Some “reverse” fittings canceled each other out to reduce overfitting as a whole. During the Dropout process, there were two possibilities for the output value of a layer of neurons to continue to propagate downward: one was to retain the original value, and the other was to be converted to 0. It was assumed that the probability of being retained was set to *x*, and the probability of being converted to 0 was 1 − *x*, with *x*=0.5 as usual. When Dropout was used as a full-connected layer, the output of this layer can be calculated by equation (6), and the output value after Dropout can be expressed as equation (7):

(6) $$\begin{array}{c} B={\left[B_{1},B_{2},\ldots ,B_{n}\right]}^{Y}, \end{array}$$

(7) $$\begin{array}{c} B=a*g\left(vu\right), \end{array}$$

where *∗* represents the element-wise product of the output *g*(*vu*) of the fully connected layer and the binary mask vector *a*; *g*(·) stands for the activation function of the full-connected layer; *u* indicates the input value vector of the full-connected layer; *v* represents the weight matrix of *z* × *c*. Besides, the length of the binary mask vector *a* is *z*, and each of the elements in *a* corresponds to a Bernoulli distribution with a statistic of *c*.

Due to the shortcomings in the fitting effect of a single algorithm, this research aimed to integrate the ensemble learning (EL) algorithm on this basis to systematically reduce the generalization error of the model through the integration of multiple models. The Extreme Gradient Boosting (XGBoost) algorithm in the EL algorithm was brought into the model. The classification and regression trees (CART) were adopted by the XGBoost algorithm as the weak classification item. In addition, its loss function expression is shown in equation (8), and the regular term expression is presented in equation (9):

(8) $$\begin{array}{c} Q=\sum_{j=1}^{m}n\left(k_{j},{\hat{k}}_{j}\right)+\sum_{c=1}^{C}\Omega \left(g_{c}\right), \end{array}$$

(9) $$\begin{array}{c} \Omega \left(g_{c}\right)=\alpha \;D+\frac{1}{2}\lambda \sum_{i=1}^{D}\omega_{i}^{2}, \end{array}$$

where $n\left(k_{j},{\hat{k}}_{j}\right)$ stands for the training error of the sample *y*_*j*_, $k_{j},{\hat{k}}_{j}$ represent the predicted value and actual value of the sample *y*_*j*_ in sequence, Ω(*g*_*c*_) means the regular term of the *c*-th CART tree, *D* indicates the number of leaf nodes of the CART tree, _*ω*_^*i*^ stands for the weight of the corresponding leaf node, and *α*, *λ* means the penalty coefficients, which are both constants.

The objective function expression of the model after the *r*-th round of iteration can be expressed as follows:

(10) $$\begin{array}{c} Q^{r}≃\sum_{j=1}^{m}n\left[k_{j},{\hat{k}}^{\left(r-1\right)}+g_{r}\left(y_{j}\right)\right]+\sum_{c=1}^{C}\Omega \left(g_{c}\right)+U, \end{array}$$

where *g*_*r*_(*y*_*j*_) represents the added *r*-th CART tree and *U* stands for the complexity of the previous tree *r* − 1, which is a constant. Then, equation (10) was expanded into Taylor's equation to get the approximate objective function, which is expressed as

(11) $$\begin{array}{c} Q^{r}≃\sum_{j=1}^{m}n\left[k_{j},{\hat{k}}^{\left(r-1\right)}+f_{j}g_{r}\left(y_{j}\right)+\frac{1}{2}s_{j}g_{r}^{2}\left(y_{j}\right)\right]+\sum_{c=1}^{C}\Omega \left(g_{c}\right)+U. \end{array}$$

Besides, *f*_*j*_ represents the first derivative of $n\left(k_{j},{\hat{k}}^{\left(r-1\right)}\right)$ and *s*_*j*_ stands for the second derivative of $k_{j},{\hat{k}}^{\left(r-1\right)}$. The final objective function could be obtained through simplified integration, which can be expressed

(12) $$\begin{array}{c} Q^{r}≃\sum_{i=1}^{D}n\left[\left(\sum_{j\in G_{i}}f_{j}\right)\omega_{i}+\frac{1}{2}\left(\sum_{j\in G_{i}}s_{j}+\lambda \right)\omega_{i}^{2}\right]+\alpha D. \end{array}$$

The optimal weight for the minimum objective function could be obtained by taking the partial derivative of *Q*^*r*^ concerning *ω*_*i*_ and setting it equal to 0. Besides, its expression is shown as follows:

(13) $$\begin{array}{c} \omega_{i}^{*}=\frac{\sum_{j\in G_{i}}f_{j}}{\sum_{j\in G_{i}}s_{j}+\lambda }. \end{array}$$

Equation (12) was incorporated into equation (13) to attain the optimal value of the objective function, which is expressed as

(14) $$\begin{array}{c} Q^{e}=-\frac{{\left(\sum_{j\in G_{i}}f_{j}\right)}^{2}}{2\left(\sum_{j\in G_{i}}s_{j}+\lambda \right)}+\alpha D. \end{array}$$

When the nodes of the subtree were split, the reduction value of the loss function after each branch node, namely, the gain, should be defined, which can be expressed as

(15) $$\begin{array}{c} \varsigma =\frac{1}{2}\left[\frac{{\left(\sum_{j\in G_{F}}f_{j}\right)}^{2}}{\left(\sum_{j\in G_{F}}s_{j}+\lambda \right)}+\frac{{\left(\sum_{j\in G_{i}}f_{j}\right)}^{2}}{\left(\sum_{j\in G_{Y}}s_{j}+\lambda \right)}-\frac{{\left(\sum_{j\in G_{i}}f_{j}\right)}^{2}}{\left(\sum_{j\in G_{i}}s_{j}+\lambda \right)}\right]-\alpha , \end{array}$$

where *G*_*F*_, *G*_*Y*_ represent the set of split nodes on the left and right sides of the classification regression tree, respectively; ∑_*j*∈*G*_*F*__*f*_*j*_ and ∑_*j*∈*G*_*F*__*s*_*j*_ stand for the sum of the statistics of the first-order gradient and the second-order gradient of the left node on the loss function, respectively. Under the guidance of the greedy algorithm, there was iteration from one leaflet, and branches were continuously added to the tree. Equation (15) was employed to calculate the gain value of each output feature at its split point, and the best split point corresponded to the highest gain value.

### 2.4. Evaluation of MRI Image Segmentation Effect Based on Deep Learning Processing

To measure the advantages and disadvantages of this algorithm, the evaluation indicators were adopted, including accuracy, precision, sensitivity, specificity, receiver operating characteristic curve (ROC), and area under the curve (AUC). In this study, the release of diagnostic targeted drugs in MRI images was correctly defined as *A*, and the release error was defined as *B*. The actual targeted drug release was correctly defined as *C*, and the release error was defined as *D*. Accuracy referred to the proportion of correct MRI image determination samples in the total number of samples, as shown in equation (16). Precision, also known as the precision ratio, represented the proportion of actual targeted release correct samples in the total samples, as shown in equation (17). Sensitivity, also known as recall rate, represented the proportion of correct samples determined by MRI images among all correct samples in actual targeted release, as shown in equation (18). Specificity meant the proportion of targeted release error samples judged by MRI in all actual targeted release error samples, as shown in equation (19):

(16) $$\begin{array}{c} \text{accuracy}=\frac{A+D}{A+B+C+D}\times 100\%, \end{array}$$

(17) $$\begin{array}{c} \text{precision}=\frac{A}{A+C}\times 100\%, \end{array}$$

(18) $$\begin{array}{c} \text{sensitivity}=\frac{A}{A+B}\times 100\%, \end{array}$$

(19) $$\begin{array}{c} \text{specificity}=\frac{D}{C+D}\times 100\%. \end{array}$$

### 2.5. MRI Image Tracking for Targeted Controlled Release of Doxorubicin Nanopreparation

After 6 hours, 12 hours, and 24 hours of taking doxorubicin nanopreparation, all the patients underwent MRI examinations. The targeted controlled release of doxorubicin nanopreparation was observed by MRI image characteristics. The patients from the two groups were treated with different MRI image analysis methods, and the analysis results were summarized and compared with the real results to analyze the application value of the two analysis methods in image tracking of the targeted release of doxorubicin nanopreparation in patients with liver cancer.

### 2.6. Statistical Methods

The test data processing was carried out using SPSS19.0 statistical software, and the measurement data were expressed as mean ± standard deviation. Besides, the comparison of the means between each group was carried out by the *t*-test, the count data were represented by percentage (%), and the *χ*^2^ test was used. In addition, *p* < 0.05 indicated that the difference was statistically substantial.

## 3. Results

### 3.1. Morphology Observation of Doxorubicin Nanopreparation under a Transmission Electron Microscope

**Figure 2:**
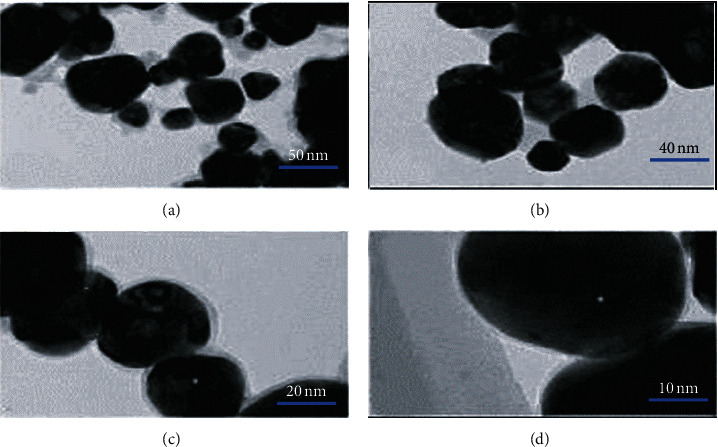
Transmission electron microscope images of doxorubicin nanopreparation. (a–d) The electron micrographs of the doxorubicin nanoformulation under the viewing angle of 50 nm, 40 nm, 20 nm, and 10 nm in turn.

Figure 2 shows a transmission electron micrograph of doxorubicin nanopreparation. It was found that the doxorubicin nonionic surfactant vesicles were spherical, smooth-edged, and uniform in size. Furthermore, the measured average particle size was 67.89 ± 24.76 nm.

### 3.2. Analysis of the Macroscopic Features of MRI Images of Patients with Liver Cancer

**Figure 3:**
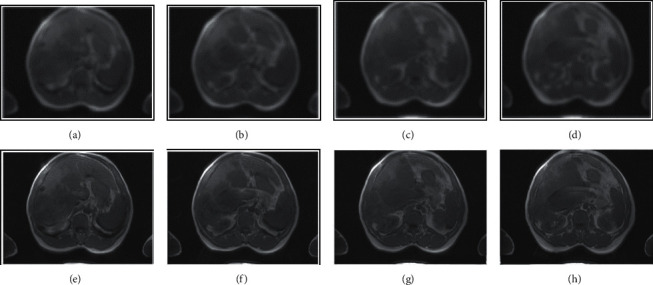
MRI images of a 55-year-old male patient with liver cancer. (a–d) *T*1, *T*2, *T*1*W*1, and *T*2*W*2 phase maps processed by traditional CNN MRI image segmentation algorithm; (e–h) *T*1, *T*2, *T*1*W*1, and *T*2*W*2 phase maps processed by the upgraded 3D CNN-based MRI image segmentation algorithm.

Figure 3 is an MRI image of a 60-year-old male patient, suggesting that the MRI image signal of liver cancer patients was unevenly distributed and diffuse compared with the uniform signal of the normal liver. Some liver cancers had envelopes, some were small nodules, and some were large masses. It indicated that there were different manifestations on the enhanced image, the fat pressure image, the *T*1 image, and the *T*2 image. Therefore, the characteristics of liver cancer could be identified by first looking at the *T*2 image, finding the difference between it and the surrounding liver. Then, the lesion could be identified by examining the enhanced *T*2*W*2 image. Compared with the traditional CNN segmentation algorithm, the MRI image segmentation processed by the 3D CNN-based segmentation algorithm was clearer, which could more accurately identify the focal sites of liver cancer patients and observe the release of nanoagents in the disease target.

### 3.3. Analysis on Imaging Indicators of MRI Image Segmentation Algorithm Based on CNN

**Figure 4:**
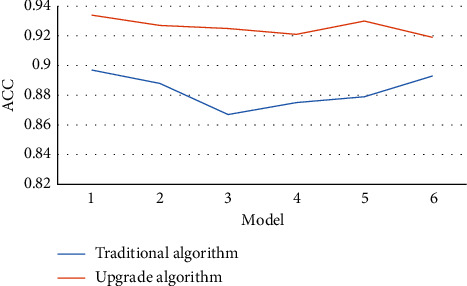
Comparison of MRI image segmentation accuracy of the two algorithms. *Note.*^*∗*^ indicates *p* < 0.05 compared with the traditional algorithm.

**Figure 5:**
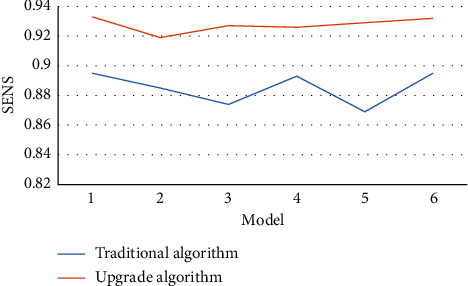
Comparison of MRI image segmentation sensitivity of the two algorithms.

**Figure 6:**
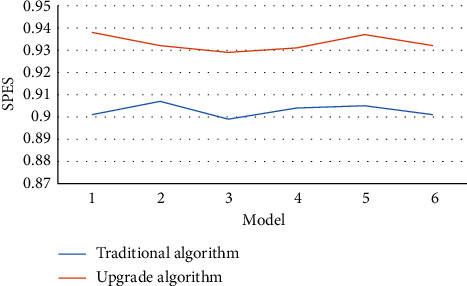
Comparison of MRI image segmentation specificity of the two algorithms.

**Figure 7:**
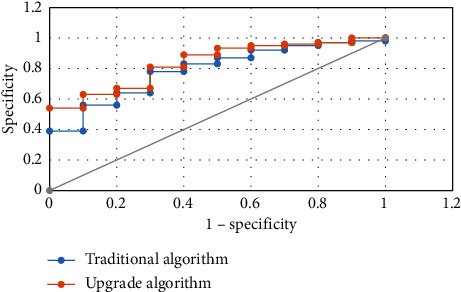
Comparison of the AUC of MRI image segmentation under two algorithms.

Figures 4–7 mean the comparison of the accuracy, sensitivity, specificity, and AUC of MRI image segmentation of traditional CNN MRI image segmentation algorithm. Compared with the traditional CNN segmentation algorithm, the 3D CNN-based MRI image segmentation algorithm had obvious advantages in accuracy, sensitivity, specificity, and AUC indicators, and the difference was statistically marked, indicating that the upgraded algorithm could further accurately segment the MRI images of liver cancer patients based on traditional algorithms, thereby enhancing the application value of MRI images.

### 3.4. Targeted Release of Doxorubicin Nanopreparation at Different Periods

**Figure 8:**
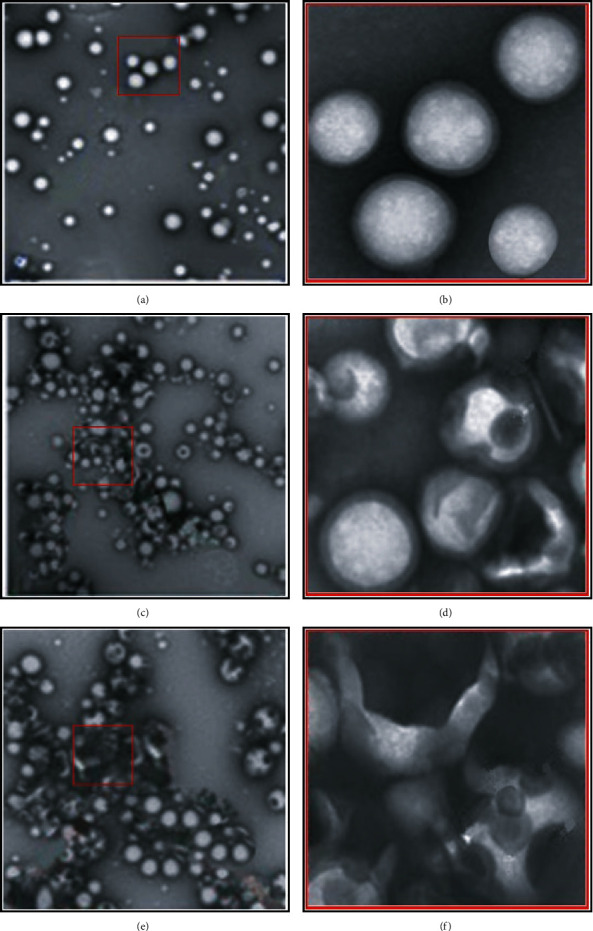
Comparison of the targeted release degree of doxorubicin nanopreparation at different periods. (a, b) The morphological diagrams after 6 hours of taking doxorubicin nanopreparation, and (b) partially enlarged diagram of (a); (c, d) the morphological diagrams of doxorubicin nanopreparation after taking 12 hours, and (d) the partially enlarged diagram of (c); (e, f) the morphological diagrams of the doxorubicin nanopreparation after 12 hours of administration, and (f) the partially enlarged diagram of (e).

Figure 8 shows the targeted release of doxorubicin observed under MRI images processed by the 3D CNN-based MRI image segmentation algorithm at different periods. When all patients took the doxorubicin nanopreparation, the drug was still in free form 6 hours after it reached the target site of the lesion and started to appear local dissolution of the doxorubicin-encapsulated nonionic surfactant vesicles. After 24 hours, the doxorubicin nanopreparation was released at the target site of the lesion.

### 3.5. Comparison on Results between Groups of MRI-Guided Targeted Controlled Release of Doxorubicin Nanopreparation

**Figure 9:**
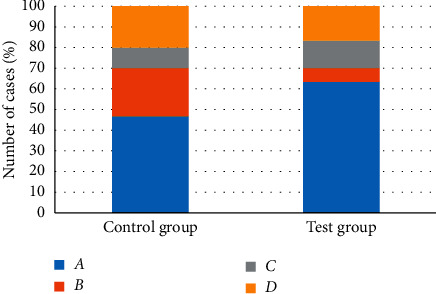
Comparison of the determination of the targeted release of doxorubicin nanopreparation between the two groups of patients. *Note. A* indicates that the targeted release of the nanopreparation was correct based on MRI images and real conditions; *B* means that the targeted release of the nanopreparation was correct by the MRI image, but the target release of the nanopreparation was incorrect in the real situation; *C* shows that the targeted release of the nanopreparation was determined by the MRI images, while the targeted release of the nanopreparation was correct in the real situation; and *D* reveals that the targeted release of the nanopreparation was determined by the MRI image and the real situation.

**Figure 10:**
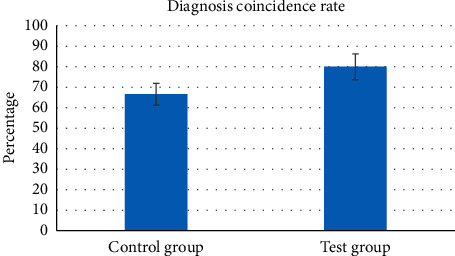
Comparison of the coincidence rate of targeted release determination between the two groups of patients. *Note.*^*∗*^ means that the target release determination accuracy rate of the experimental group was significantly higher than that of the control group, and the difference was statistically marked (*p* < 0.05).

Figure 9 shows the judgment of the results of the targeted release of doxorubicin nanoformulations under the guidance of different MRI image diagnosis methods for the two groups of patients. It was found that the number of patients from the experimental group whose diagnosis results were consistent with the actual situation was 24. The number of patients from the control group whose diagnosis results were in line with the actual situation was 19, and the difference between the two groups was statistically substantial (*p* < 0.05). The diagnostic coincidence rate of the two methods was calculated by comparing with the real data obtained at the later stage of treatment.  Figure 10 shows the specific information, revealing that compared with the control group, the accuracy of the determination of the release of doxorubicin nanopreparation in the experimental group was significantly improved, as high as 80 ± 6.25%, which was higher hugely than that of the control group (66.6 ± 5.32%), and the difference was statistically obvious (*p* < 0.05).

## 4. Discussion

At present, targeted drug therapy is one of the standard treatments for patients with advanced liver cancer [17]. This method designs targeted therapeutic drugs for the identified carcinogenic sites at the cellular and molecular levels. After the drug enters the body, it can specifically select the carcinogenic site to combine with the properties, specifically killing the tumor cells without affecting the normal tissue cells around the tumor. Therefore, this method has the advantages of high efficiency and strong specificity [18].

Currently, doxorubicin is often used in targeted drug therapy for liver cancer patients, and there have been several reports on the development of nanotargeted drugs for this drug [18]. However, MRI image-guided analysis of the targeted controlled release process of doxorubicin nanoagents in vivo has not been reported. Therefore, this study hoped to introduce the CNN-based MRI image segmentation algorithm and apply it to the guided analysis of MRI images in the targeted controlled release of doxorubicin nanomaterials to analyze the imaging analysis effect of this algorithm on the targeted treatment of liver cancer with doxorubicin nanopreparation. The results of this study showed that compared with the traditional CNN MRI image segmentation effect, the upgraded 3D CNN-based MRI image segmentation effect was better, with significant improvement in the accuracy, sensitivity, and specificity indicators, and the differences were all statistically marked (*p* < 0.05). This was similar to the research results of Fang et al. [19], indicating that the image segmentation algorithm based on CNN could improve the quality of MRI images very well. In the monitoring of the targeted drug therapy of doxorubicin nanopreparations for liver cancer patients, it was found that the MRI images of liver cancer patients processed by a 3D CNN-based MRI image segmentation algorithm could be more intuitively observed and guided to accurately arrive at the target of liver cancer to accurately kill liver cancer cells and optimize the targeted therapy process of liver cancer.

## 5. Conclusion

In this study, an upgraded version of the 3D neural network MRI image segmentation algorithm was designed based on the fully CNN, which was applied to the MRI image analysis of the targeted controlled release of doxorubicin nanopreparation in the experimental group of 40 liver cancer patients. The results found that compared with the traditional CNN MRI image segmentation algorithm, the neural network upgrade algorithm was hugely optimized in terms of image segmentation accuracy, sensitivity, specificity, and AUC; this algorithm was effective in MRI image guidance and tracking in the targeted therapy of doxorubicin nanopreparation in clinical liver cancer patients. However, the selection of patient samples in this study is limited and the source is single, which make this study not analyze the MRI image characteristics of targeted therapy of doxorubicin nanopreparation in patients with different types of liver cancer. In the future, it is considered to increase the sample size of liver cancer patients and further adopt the analysis method of multicenter cooperation for the study. All in all, the results of this study can provide a good theoretical basis for the clinical application of the MRI image segmentation algorithm of CNN in the targeted therapy of doxorubicin nanopreparation in patients with liver cancer.

## Data Availability

The data used to support the findings of this study are available from the corresponding author upon request.
